# Borrowing information across patient subgroups in clinical trials, with application to a paediatric trial

**DOI:** 10.1186/s12874-022-01539-3

**Published:** 2022-02-20

**Authors:** Rebecca M. Turner, Anna Turkova, Cecilia L. Moore, Alasdair Bamford, Moherndran Archary, Linda N. Barlow-Mosha, Mark F. Cotton, Tim R. Cressey, Elizabeth Kaudha, Abbas Lugemwa, Hermione Lyall, Hilda A. Mujuru, Veronica Mulenga, Victor Musiime, Pablo Rojo, Gareth Tudor-Williams, Steven B. Welch, Diana M. Gibb, Deborah Ford, Ian R. White, and the ODYSSEY Trial Team

**Affiliations:** 1 Medical Research Council Clinical Trials Unit at University College London, 90 High Holborn, London, WC1V 6LJ UK; 2 Department of Paediatric Infectious Diseases, Great Ormond Street Hospital for Children NHS Foundation Trust, London, UK; 3 UCL Great Ormond Street Institute of Child Health, London, UK; 4 King Edward VIII Hospital, Durban, South Africa; 5 Department of Paediatrics and Child Health, University of KwaZulu Natal, Durban, South Africa; 6 Makerere University- Johns Hopkins University Research Collaboration, Kampala, Uganda; 7 Family Center for Research With Ubuntu, Department of Paediatrics and Child Health, Tygerberg Hospital and Stellenbosch University, Cape Town, South Africa; 8 PHPT/IRD UMI174, Faculty of Associated Medical Sciences, Chiang Mai University, Chiang Mai, Thailand; 9 Department of Molecular & Clinical Pharmacology, University of Liverpool, Liverpool, UK; 10 Joint Clinical Research Centre, Kampala, Uganda; 11 Joint Clinical Research Centre, Mbarara, Uganda; 12 Department of Paediatric Infectious Diseases, Imperial College Healthcare NHS Trust, London, UK; 13 University of Zimbabwe Clinical Research Centre, Harare, Zimbabwe; 14 University Teaching Hospital, Lusaka, Zambia; 15 Department of Paediatrics and Child Health, School of Medicine, College of Health Sciences, Makerere University, Kampala, Uganda; 16 Hospital, 12 de Octubre, Madrid, Spain; 17 Imperial College, London, UK; 18 Department of Paediatrics, Birmingham Chest Clinic and Heartlands Hospital, University Hospitals Birmingham, Birmingham, UK

**Keywords:** Paediatric trials, Subgroups, Small samples, Bayesian analysis, Borrowing information

## Abstract

**Background:**

Clinical trial investigators may need to evaluate treatment effects in a specific subgroup (or subgroups) of participants in addition to reporting results of the entire study population. Such subgroups lack power to detect a treatment effect, but there may be strong justification for borrowing information from a larger patient group within the same trial, while allowing for differences between populations. Our aim was to develop methods for eliciting expert opinions about differences in treatment effect between patient populations, and to incorporate these opinions into a Bayesian analysis.

**Methods:**

We used an interaction parameter to model the relationship between underlying treatment effects in two subgroups. Elicitation was used to obtain clinical opinions on the likely values of the interaction parameter, since this parameter is poorly informed by the data. Feedback was provided to experts to communicate how uncertainty about the interaction parameter corresponds with relative weights allocated to subgroups in the Bayesian analysis. The impact on the planned analysis was then determined.

**Results:**

The methods were applied to an ongoing non-inferiority trial designed to compare antiretroviral therapy regimens in 707 children living with HIV and weighing ≥ 14 kg, with an additional group of 85 younger children weighing < 14 kg in whom the treatment effect will be estimated separately. Expert clinical opinion was elicited and demonstrated that substantial borrowing is supported. Clinical experts chose on average to allocate a relative weight of 78% (reduced from 90% based on sample size) to data from children weighing ≥ 14 kg in a Bayesian analysis of the children weighing < 14 kg. The total effective sample size in the Bayesian analysis was 386 children, providing 84% predictive power to exclude a difference of more than 10% between arms, whereas the 85 younger children weighing < 14 kg provided only 20% power in a standalone frequentist analysis.

**Conclusions:**

Borrowing information from a larger subgroup or subgroups can facilitate estimation of treatment effects in small subgroups within a clinical trial, leading to improved power and precision. Informative prior distributions for interaction parameters are required to inform the degree of borrowing and can be informed by expert opinion. We demonstrated accessible methods for obtaining opinions.

**Supplementary Information:**

The online version contains supplementary material available at 10.1186/s12874-022-01539-3.

## Background

Phase III clinical trials evaluate the effectiveness and safety of medical interventions in a chosen patient population. For biological or operational reasons, investigators may choose to evaluate effectiveness and safety in a specific subgroup (or subgroups) of patients within the main trial population, in addition to reporting on the whole population. Subgroups of interest may be defined by patient characteristics, disease characteristics or biomarkers. A subgroup might represent a small population that merits being studied in a standalone trial, but for which sufficient recruitment would be difficult, for example paediatric sub-populations or rare disease subtypes. Basket trials are designed specifically to study multiple patient subgroups: a single treatment is delivered to subtypes of patients with the same disease, aiming to draw conclusions about effectiveness within rather than across subtypes [1, 2].

Sample sizes within subgroups are usually small and tend not to provide sufficient power for evaluating treatment effects in a standalone analysis. However, if the treatment effect in a subgroup is expected to be similar to that in the remainder of the trial population, there is justification for borrowing information across patient subgroups using Bayesian methods. Several authors have recommended using a shrinkage approach to borrowing across multiple subgroups, in which treatment effects for separate subgroups are assumed exchangeable and drawn from a common random distribution, with or without stratification by subgroup characteristics [3–6]. Using this approach, the treatment effect for any single subgroup is informed by the effects observed in other subgroups and pulled towards the overall average treatment effect.

In this paper, our focus is to evaluate a treatment effect in a pre-defined subgroup of interest, which we call the target subgroup, while borrowing information from a separate patient subgroup, using a Bayesian analysis. In this setting, a hierarchical shrinkage approach would not be appropriate since this requires estimation of the variability across subgroups, which is not possible with only two subgroups. Instead we use a simple model to borrow information from one patient subgroup for the other. In addition, we adjust the weight given to the borrowed information based on clinical opinion about the similarity of treatment effects in the two subgroups. An advantage of using a simple rather than complex model is that uncertainty about similarity of treatment effects has direct correspondence to weights allocated to each subgroup, with the interpretation of the model being easily communicated to clinicians.

### Motivating example

The ODYSSEY (PENTA-20) trial is an open-label, randomised trial evaluating the efficacy and safety of once daily dolutegravir-based antiretroviral therapy (ART) versus standard of care (SOC) in children and adolescents living with HIV and starting first- or second-line ART (ISRCTN91737921) [7]. A non-inferiority design was used with the trial powered to exclude an absolute difference of more than 10% in combined clinical and virological failure rates between the dolutegravir (DTG) and SOC arms by 96 weeks, assuming an overall failure rate in both arms of 18%. Failure was defined as the first occurrence of any of: (1) insufficient virological response at week 24 with treatment switch to second-/third-line; (2) virological failure (two consecutive viral load measures ≥ 400 c/ml with the first at/after week 36); (3) new or recurrent AIDS defining event (WHO 4) or severe WHO 3 event or (4) all-cause death. The main trial recruited 707 children weighing ≥ 14 kg (age range 2.9–18 years; 96% ≥ 6 years) between September 2016 and June 2018; 85 children weighing 3 to < 14 kg (range 0.13–5.9 years; 89% < 3 years) were subsequently recruited between July 2018 and August 2019. A lead-in pharmacokinetics (PK) sub-study in the DTG arm was conducted to provide data on dosing in young children. For convenience, we refer in this paper to the two groups of children as “older” and “younger” as well as “weighing ≥ 14 kg” and “weighing < 14 kg”.

The 96 week follow-up of the children recruited weighing < 14 kg will be complete approximately 12 months after the 96 week follow-up of the children weighing ≥ 14 kg. The ODYSSEY team opted not to delay presentation of the results from the main trial participants weighing ≥ 14 kg. Sample size in the younger children is small (*n* = 85) and therefore a standalone analysis of their data will not be adequately powered to test the difference in failure rates. Since treatment effects between the DTG and SOC arms may be similar in the older and younger children, there is an opportunity to borrow information from the older children when analysing the younger children, through a Bayesian analysis.

If treatment effects in older and younger children were considered identical, it would be appropriate for conclusions about the treatment effect in younger children to be based on a combined analysis. The much larger sample of older children would dominate this analysis, receiving ~ 90% of the weight. If, as is more likely, treatment effects were considered similar but not identical, it would be appropriate to borrow partial rather than full information from the older children to estimate the treatment effect in the younger children. The amount of information borrowed should reflect the assumed similarity of the true treatment effects. Reasons for treatment effects to differ might include: differences in the range and formulations of ART drugs available in the SOC arm, differing adherence to treatment (influenced by tolerability and acceptability of drugs and whether or not caregivers administer doses), differences in antiretroviral exposure by weight or age and differences in viral dynamics. The ODYSSEY team decided to use elicitation methods [8] to obtain clinical opinion on the similarity of these treatment effects, before the main trial results were known, to inform the weighting of data from older children in a planned Bayesian analysis of the younger children.

## Methods

### Model

Our aim was to estimate a treatment effect ${\theta }_{1}$ in a target subgroup of patients within a trial (e.g. the younger children in ODYSSEY), while borrowing information obtained from a larger subgroup of patients within the same trial. Suppose that data from the target subgroup provide an estimate ${y}_{1}$ of ${\theta }_{1}$ with standard error ${\sigma }_{1}$, and we assume:

1 $${y}_{1}\sim N\left({\theta }_{1},{\sigma }_{1}^{2}\right)$$

Suppose also that data from the larger subgroup (e.g. the older children in ODYSSEY) provide the following estimate ${y}_{0}$ of treatment effect ${\theta }_{0}$ in the larger subgroup, with standard error ${\sigma }_{0}$:

2 $${y}_{0}\sim N\left({\theta }_{0},{\sigma }_{0}^{2}\right)$$

We introduce an interaction parameter $\delta$ to describe the relationship between treatment effects in the two subgroups: ${\theta }_{1}={\theta }_{0}+\delta$. Elicitation can be used to obtain opinions about likely values for $\delta$, which represents an interaction between treatment and subgroup. In the ODYSSEY trial, the treatment effect of interest is a risk difference and $\delta$ is a difference in risk differences. We assume a normal distribution for $\delta$ and use elicited opinion to inform choice of the standard deviation ${\sigma }_{\delta }$:

3 $$\delta \sim N\left(0,{\sigma }_{\delta }^{2}\right)$$

We choose a mean of 0 for the distribution for $\delta$, rather than using expert opinion to inform this assumption, because we wanted to use clinical opinion to inform the weight given to data from the larger subgroup but not to directly alter the location of the estimate for the smaller subgroup. We choose to specify a flat normal prior for ${\theta }_{0}$, since we do not want to introduce any prior belief about the magnitude of the treatment effect:

4 $${\theta }_{0}\sim N\left(0,{10}^{6}\right)$$

Under a framework proposed by Hobbs et al., the resulting prior for ${\theta }_{1}$ is a location commensurate prior with commensurability parameter ${\sigma }_{\delta }$ [9], where a commensurate prior is a prior distribution describing the extent to which a parameter in a new study varies around the corresponding parameter in a previous study or studies. Larger values for ${\sigma }_{\delta }$ represent greater uncertainty about the magnitude of the interaction and correspond to the larger subgroup of patients contributing less information to the target subgroup analysis. A value ${\sigma }_{\delta }=0$ represents certainty that ${\theta }_{1}={\theta }_{0}$ and would result in the two subgroups being combined.

In a combined analysis using data sets from both subgroups, the treatment effect in the target subgroup is estimated as follows:

5 $${\theta }_{1}|{y}_{1},{y}_{0},\delta \sim N\left(\frac{{y}_{1}/{\sigma }_{1}^{2}+{y}_{0}/\left({\sigma }_{0}^{2}+{\sigma }_{\delta }^{2}\right)}{1/{\sigma }_{1}^{2}+1/\left({\sigma }_{0}^{2}+{\sigma }_{\delta }^{2}\right)},\frac{1}{1/{\sigma }_{1}^{2}+1/\left({\sigma }_{0}^{2}+{\sigma }_{\delta }^{2}\right)}\right)$$

We note that ${\sigma }_{1}^{2}$ and ${\sigma }_{0}^{2}$ are assumed fixed and known, as estimated from the data, and no allowance is made for their uncertainty. The motivation for choosing a simple model and assuming normal distributions is that the variance of $\delta$ has a direct correspondence to the relative weights allocated to the two subgroups of patients in the analysis. This simplifies communicating to clinicians how uncertainty about $\delta$ affects the results of the Bayesian analysis. In this analysis, the relative weight given to the larger subgroup in estimation of the treatment effect in the target subgroup is:

6 $$\frac{1}{{\sigma }_{0}^{2}+{\sigma }_{\delta }^{2}}/\left(\frac{1}{{\sigma }_{1}^{2}}+\frac{1}{{\sigma }_{0}^{2}+{\sigma }_{\delta }^{2}}\right)$$

Derivations of formulae (5) and (6) are provided in supplementary material (Additional file 1), together with a mathematical description of the model.

### Elicitation methods

Expert opinion was sought on the interaction parameter $\delta$ representing the difference between the risk difference in the younger children weighing < 14 kg and the risk difference in the older children weighing ≥ 14 kg.

A pilot elicitation study was carried out in which four methods were implemented and evaluated by five experts with paediatric or statistical expertise (authors AB,DF,DMG,CLM,AT). Feedback informed us that experts found it helpful to be asked the same question in multiple ways, and then to be asked to moderate their answers, because this clarified their thought process. This approach allowed us to check and discuss the coherency of the experts’ statements about their uncertainty [10]. We therefore decided to include three methods in our subsequent elicitation exercise, chosen on the basis of being well understood. The fourth method involved eliciting an estimate together with an inter-quartile range which was not easily communicated or understood.

In the final elicitation procedure, thirteen experts practising as paediatric HIV clinicians (including eleven ODYSSEY trial investigators and one member of the ODYSSEY Endpoint Review Committee) were asked to provide initial answers under each of two methods (stages 1 and 2 below) and were then asked to moderate their answers by providing an answer under a third method (stage 3). The stages of the elicitation procedure were identical across experts.

#### Stage 1

Experts were asked to assume that data were available from a very large trial comparing the two arms, DTG to SOC regimens, in older children weighing ≥ 14 kg, in which the failure rate by 96 weeks in the SOC arm was 18% and the treatment difference in failure rates was estimated as 5% in favour of the DTG arm. They were asked to suppose that the trial was so large that sampling variability was close to zero and the observed estimate was very close to the true treatment effect in older children. This assumption was discussed to ensure that experts understood our focus was on uncertainty arising from imperfect knowledge rather than uncertainty arising from sampling variation [8]. We note that the assumed values were hypothetical.

Opinion was elicited on the risk difference in younger children recruited weighing < 14 kg (age under approximately 3 years but all > 4 weeks of age), rather than directly on the difference in risk differences. In order to elicit a range for the experts’ uncertainty about the risk difference in younger children, we asked them to consider what size of true difference in younger children would surprise them, first in the direction of more extreme risk differences favouring DTG and then in the opposite direction. These values formed their uncertainty range. Eliciting uncertainty ranges provides a mean and variance for each expert’s probability distribution, under the assumption of normality; people have been shown to perform better when assessing intervals rather than variances, and it is preferable to avoid eliciting a mean value first to avoid anchoring effects [8, 10].

Next, experts were asked to assign a probability to their chosen uncertainty range, to represent how likely they believed it was that the true risk difference in younger children was included. They were asked to think about placing 100 counters either inside or on either side of the range, and were given an opportunity to use physical counters or draw their distribution to help visualise their probability beliefs, following a “bins and chips” approach to assigning probabilities to intervals (Figure S2) [11, 12].

#### Stage 2

The second stage was identical to the first stage, except for the risk difference assumed in older children. Here, experts were asked to assume that failure rates of 18% by 96 weeks had been observed in both arms in a very large trial comparing DTG to SOC in children recruited weighing ≥ 14 kg.

#### Stage 3

Experts were asked to consider the weight allocated to the data from older children if conclusions about the risk difference in younger children were based on a combined analysis of both subgroups. They were informed that the data from older children would receive approximately 90% of the weight if weightings were based on sample sizes alone or 0% of the weight if these data were ignored. They were then asked how much weight they would like allocated to the data from older children. Experts were not required to provide a rationale for their beliefs, but comments on rationale were documented if mentioned. An Excel spreadsheet was provided (Figure S1 in Additional file 1) to illustrate the correspondence between weights allocated to the data from older children and beliefs about the uncertainty range for the treatment effect in younger children. Choices for the weight, assumed risk difference in older children and level of uncertainty could be altered within the spreadsheet. Experts were asked to consider their initial range choices and think about the correspondence between weight in combined analysis and uncertainty ranges, and then make a final choice of weight to be allocated to the older children’s data.

### Elicitation process

Paediatricians with expertise in HIV treatment and management, nearly all of whom were researchers enrolling children in ODYSSEY and/or other paediatric HIV studies, were invited to participate in the elicitations. As it is recommended that opinion-based prior distributions represent a breadth of opinions [13], we invited experts from several different countries. The chosen experts all had sufficient experience and knowledge to provide valid and reliable descriptions of the quantities of interest [12]. Conducting elicitations remotely would have been possible, but face-to-face elicitations were preferred because experts can find the elicitation process difficult and it is useful to have a facilitator on hand to answer questions [8, 14]. We therefore carried out the elicitation exercise alongside an international PENTA-ID Network meeting [15] in May 2019. Recruitment was still ongoing at that time and elicitations assumed that the trial would include 700 children weighing ≥ 14 kg and 80 children weighing < 14 kg. The elicitations were carried out before results from the older children were available in order that opinions were not influenced by knowledge of the results. This avoided using the data from older children twice in the Bayesian analysis. Thirteen experts participated in the elicitation exercise; 12 experts were interviewed face-to-face by RT in 1:1 meetings and one expert was interviewed by telephone (since a face-to-face meeting was not possible).

### Analysis of elicitation results

We mapped each expert’s chosen uncertainty range $\left(a,b\right)$ and corresponding probability *p* to a normal distribution. The following values were calculated for the mean $\mu$ and standard deviation $\sigma$ of the expert’s probability distribution:

7 $$\mu =\frac{a+b}{2}$$

$$\sigma =\frac{b-a}{2{\Phi }^{-1}\left(\frac{p+1}{2}\right)}$$

where $\Phi$ is the cumulative distribution function of the standard normal distribution. We present means and inter-quartile ranges from the fitted distributions rather than the original ranges chosen, in order that distributions are comparable across experts who assigned different probabilities to their ranges.

The relative weight to be allocated to evidence from the ≥ 14 kg children in Bayesian analysis of the < 14 kg children was derived from the clinical opinions elicited. To pool opinions across experts, we used the median of the weights chosen. This method of pooling was chosen in order that the pooled weight represents the opinion of a ‘typical’ expert and is not influenced by extreme opinions.

### Planned Bayesian analysis in the ODYSSEY trial

A Bayesian analysis will be reported for the children weighing < 14 kg, alongside frequentist analyses of the children weighing < 14 kg and of the whole trial population (< 14 kg and ≥ 14 kg). The analysis is expected to be performed in early 2022. The Statistical Analysis Plan was written in advance of conducting the elicitations and specifies that if at least 80% of the experts chose weights within a 30% absolute range, the Bayesian analysis will be reported as the primary analysis; alternatively, if less than 80% of the experts chose weights within a 30% absolute range, the Bayesian analysis would be reported as a secondary analysis and the frequentist analysis of the < 14 kg children would be reported as the primary analysis of these data. The range threshold of 30% was chosen on the basis of how much variation in opinion among the clinical experts was considered acceptable.

## Results

### Descriptive analysis of elicitation results

**Fig. 1:**
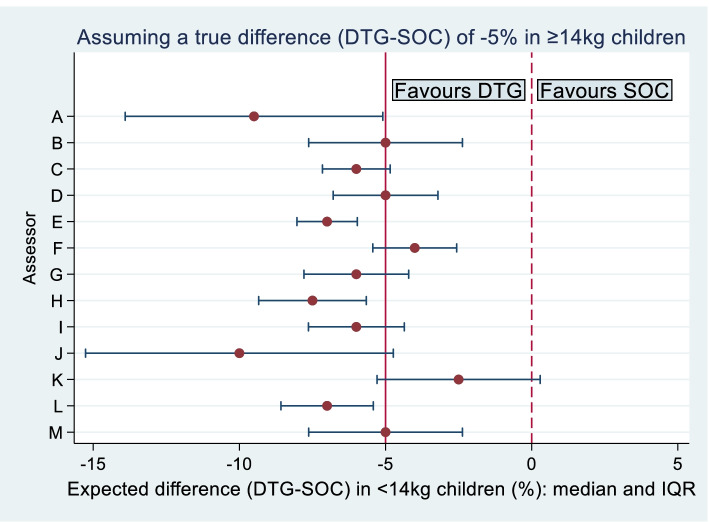
Experts’ opinions about the true treatment difference in 96-week failure rates in children weighing < 14 kg, assuming a 5% treatment difference in favour of DTG in children weighing ≥ 14 kg (stage 1): medians and inter-quartile ranges of fitted probability distributions

Figure 1 summarises the experts’ opinions from stage 1, about the difference in failure rates in children weighing < 14 kg in the DTG arm vs the SOC arm, when assuming the true treatment difference in children weighing ≥ 14 kg is $-5\%$ (DTG $-$ SOC). On average, experts expected the treatment benefit to be slightly greater in children weighing < 14 kg: the median of mean values was $-6\%$, with inter-quartile range $-7\%$ to $-5\%$. The probabilities assigned to their chosen uncertainty ranges ranged from 60 to 98%, with a median value of 90%. Three experts commented on their rationale for expecting treatment benefit to be greater in younger children; reasons given were that (i) adherence is higher in younger children than older children (carers give /supervise treatment in young children, whereas adolescents are known to be less adherent) and (ii) the DTG comparator formulations (SOC regimens) available for young children (i.e. mostly lopinavir/ritonavir pellets) are less well tolerated than SOC drugs used in older children.

**Fig. 2:**
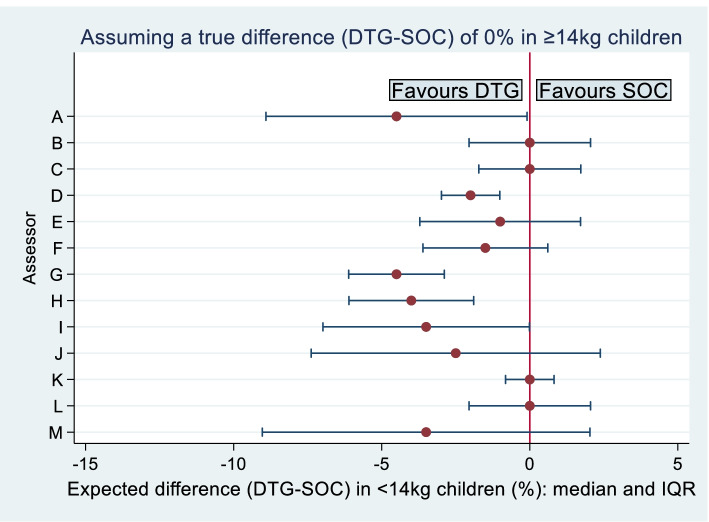
Experts’ opinions about the true treatment difference in 96-week failure rates in children weighing < 14 kg, assuming a 0% treatment difference in children weighing ≥ 14 kg (stage 2): medians and inter-quartile ranges of fitted probability distributions

Figure 2 summarises the experts’ opinions from stage 2, when assuming no difference in failure rates between DTG and SOC in children weighing ≥ 14 kg. On average, the treatment benefit was again expected to be slightly greater in children weighing < 14 kg: the median of mean values was $-2\%$, with inter-quartile range $-4\%$ to 0 $\%$. The probabilities assigned to the experts’ chosen uncertainty ranges ranged from 60% to 96%, with a median value of 90%. The opinions expressed when assuming no difference in failure rates in older children were reasonably consistent with those expressed when assuming a 5 $\%$ benefit for DTG.

**Fig. 3:**
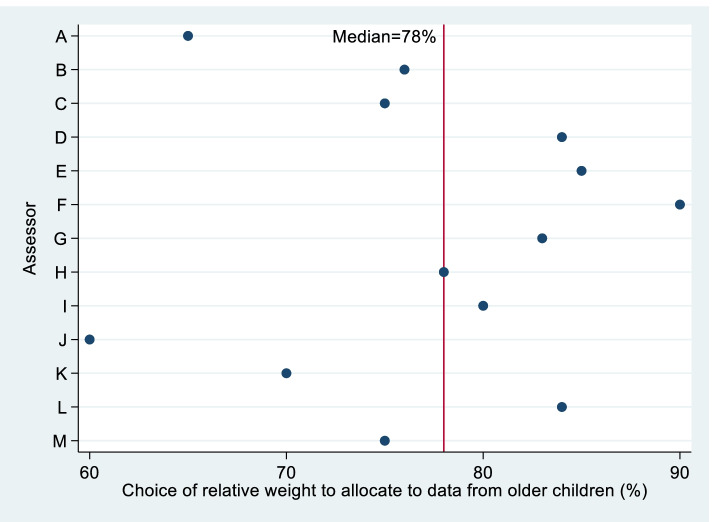
Experts’ choices for relative weight to allocate to data from children weighing ≥ 14 kg in a Bayesian analysis of the children weighing < 14 kg (stage 3)

After considering the correspondence between uncertainty ranges chosen and the relative weight that would be allocated to the data from older children in a combined analysis and reviewing their initial range choices, experts made a final choice for the relative weight (Fig. 3). It was explained that this weight rather than the initial range choices would be used in the planned Bayesian analysis. One expert (L) requested that their range choice under stage 1 be used in the analysis rather than their chosen weight, because they were more confident about their initial choice, and we therefore mapped their range to a weight.

The median of the relative weights chosen was 78%, with inter-quartile range from 75% to 84%. When assuming a $-5\%$ difference in children weighing ≥ 14 kg, a weight of 78% corresponds to a 95% range of $-12\%$ to $2\%$ for the treatment difference in children weighing < 14 kg. All experts chose weights within a 30% absolute range, meaning that the pre-specified criterion for the Bayesian analysis to be reported as the primary analysis of the < 14 kg children was met.

### Impact on analysis of ODYSSEY trial

We then explored the potential impact of incorporating evidence obtained from the older children as a prior distribution in analysis of the younger children in the ODYSSEY trial, using the final sample sizes of 707 children weighing ≥ 14 kg and 85 children weighing < 14 kg, together with hypothesized observed treatment effects. In frequentist analyses, the DTG regimen is judged non-inferior to SOC if the upper bound of the 95% confidence interval for the difference in proportions failing (DTG $-$ SOC) is less than 10%. In Bayesian analyses, the DTG regimen is judged non-inferior to SOC if the upper bound of the corresponding 95% credible interval is less than 10%.

When applying the chosen relative weight of 78%, the evidence from 707 children weighing ≥ 14 kg is given an effective sample size equivalent to 301 children in the Bayesian analysis, assuming failure rates of 18% in both arms. The total effective sample size in the Bayesian analysis is therefore 301 + 85 = 386 children. We assume flat Unif(0,1) priors for the failure rates in each treatment arm in the older children. As a measure of the power provided by the Bayesian analysis, we calculate predictive power, which is the predictive probability of obtaining a ‘significant’ result in the planned Bayesian analysis [16]. Under the decision criteria above, a significant Bayesian result is defined here as a 95% credible interval for the difference in proportions failing (DTG $-$ SOC) that has an upper bound lower than 10%. The Bayesian analysis provides 84% predictive power to exclude (at two-sided 5% significance level) a difference beyond the non-inferiority margin of 10% between the two arms, allowing for 10% loss to follow-up. For comparison, the sample of 85 children weighing < 14 kg provides 20% power in a standalone frequentist analysis.

#### Example 1

First, we consider a scenario in which the direction of the observed treatment effect differs between the older and younger children. Suppose the treatment difference in 96-week failure rates between DTG and SOC (DTG $-$ SOC) was estimated empirically in each subgroup as $-2\%$ (95% confidence interval (CI): $-8\%$ to 4 $\%$) in children weighing ≥ 14 kg and as 7 $\%$ (95% CI: $-10\%$ to 24 $\%$) in children weighing < 14 kg. Under this data scenario, the DTG regimen is judged non-inferior to SOC in children weighing ≥ 14 kg. In a standalone analysis of the small sample of 85 children weighing < 14 kg, however, the 95% confidence interval is extremely wide, with DTG not judged non-inferior.

**Fig. 4:**
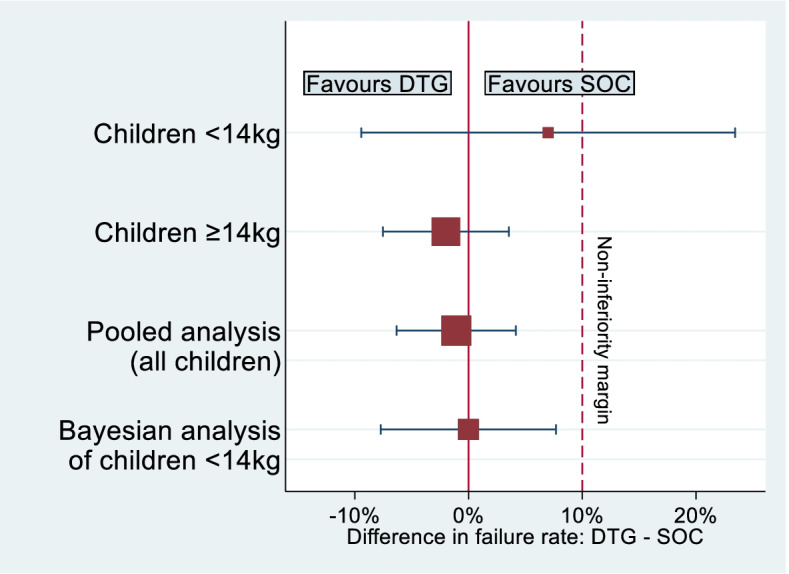
Treatment difference estimates and 95% intervals in Example 1, obtained from a pooled analysis of all children and a Bayesian analysis for children weighing < 14 kg (incorporating evidence from children weighing ≥ 14 kg as a prior distribution), together with results from separate subgroups

In a Bayesian analysis of the children weighing < 14 kg (Fig. 4), the treatment difference in failure rates (DTG $-$ SOC) is estimated from expression (5) as $0\%$ (95% credible interval (CrI): $-8\%$ to 8 $\%$) and we conclude non-inferiority for the DTG regimen in children weighing < 14 kg. The conclusion changes when the data from older children are incorporated and we would prefer to report the Bayesian analysis informed by the clinical opinions elicited, in preference to the standalone analysis. A standard pooled data analysis of both subgroups would be appropriate if the treatment differences were believed to be identical across subgroups, and produces an estimate of $-1\%$ (95% CI: $-6\%$ to 4 $\%$). As a sensitivity analysis, we also performed the Bayesian analysis using each expert’s chosen relative weight in turn. The results ranged from an estimated treatment difference of -1% (95% CI: -6% to 4%) to an estimate of 2% (95% CI: -9% to 12%).

#### Example 2

**Fig. 5:**
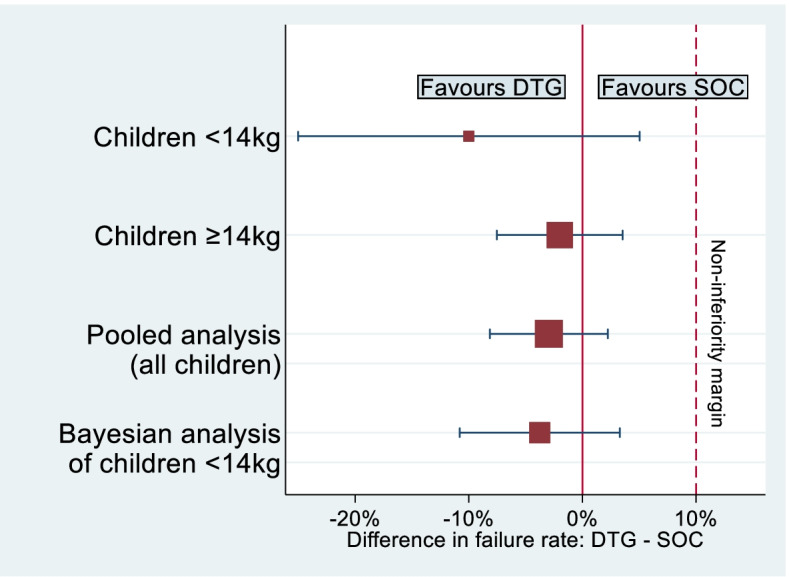
Treatment difference estimates and 95% intervals in Example 2, obtained from a pooled analysis of all children and a Bayesian analysis for children weighing < 14 kg (incorporating evidence from children weighing ≥ 14 kg as a prior distribution), together with results from separate subgroups

Next, we consider a scenario where the observed treatment effect in younger children is in the same direction but more extreme than that in older children. Suppose that the treatment difference in 96-week failure rates (DTG $-$ SOC) was estimated empirically as $-10\%$ (95% CI: $-25\%$ to $5\%$) in children weighing < 14 kg, while the observed difference in children weighing ≥ 14 kg remains $-2\%$ (95% CI: $-8\%$ to 4 $\%$) as in Example 1. Under this scenario, the DTG regimen is judged non-inferior to the SOC regimen in the small sample of children weighing < 14 kg, although the treatment difference is very imprecisely estimated. In a Bayesian analysis incorporating evidence from the children weighing ≥ 14 kg, the treatment difference is pulled back towards the null value and estimated from expression (5) as $-4\%$ (95% CrI: $-11\%$ to 3 $\%$) in favour of DTG (Fig. 5). As in Example 1, we would prefer to report the Bayesian analysis informed by clinical opinion in preference to the imprecise and more extreme standalone analysis of the younger children. A standard pooled analysis produces an estimate of $-3\%$(95% CI: $-8\%$ to 2 $\%$). As a sensitivity analysis, we also performed the Bayesian analysis using each expert’s chosen relative weight in turn. The results ranged from an estimated treatment difference of -5% (95% CI: -15% to 4%) to an estimate of -3% (95% CI: -8% to 2%).

## Discussion

We have described methods for evaluating a treatment effect in a small subgroup within a clinical trial, while borrowing information from the main trial population. In the ODYSSEY trial, clinical experts chose on average to allocate a relative weight of 78% (reduced from ~ 90% based on sample size) to data from children weighing ≥ 14 kg in a Bayesian analysis of the children weighing < 14 kg. Borrowing information from the older children leads to substantial increases in the power and precision of the analysis, in comparison with an analysis based on the younger children alone. To provide transparency for those concerned about incorporating borrowed information, we recommend reporting results from the Bayesian analysis alongside results from the standalone analysis and a conventional combined analysis.

Similar methods could be applied when borrowing information across multiple subgroups in a basket trial, to inform estimation of treatment effects in baskets insufficiently powered for a standalone analysis. In paediatric trials, there could be rationale for estimating treatment effects within multiple narrower age groups, where one or more of these include low numbers of children. In cancer trials, if a basket trial studying biologically related cancers includes some common and some rare cancers, information could be borrowed from the large baskets to inform estimation of treatment effect in the small baskets. If baskets are adequately powered, however, there is no need to borrow information. For example, the main ODYSSEY trial population of older children included two baskets (children starting first-line or second-line ART) that were adequately powered for standalone analysis. Similar methods can be used to incorporate information from adult trials when analysing paediatric trials [17, 18].

Clinical opinion was used to inform the weight given to the data from older children in analysis of the younger children, but we chose to assume a zero mean for the interaction parameter rather than using expert opinion to inform this. This means that if the set of opinions were shifted to the left or right in Fig. 1, the Bayesian analysis would be unchanged. Independent priors were assumed for the interaction parameter and the treatment effect ${\theta }_{0}$ in older children; this is supported by the similarity of opinions presented in Figs. 1 and 2, and because experts are expected to be much more certain about the interaction parameter than about ${\theta }_{0}$ a priori. When eliciting clinical opinion, we chose to use an individual elicitation rather than group elicitation approach, in order to obtain a variety of opinions and to avoid the potential problem that one or two individuals could be overly influential in the choice of a consensus distribution [8]. Carrying out individual face-to-face elicitations is time-consuming and logistically challenging when the chosen experts are from multiple countries. Using an online remote elicitation process would allow opinions to be collected from a larger number of experts [19, 20], but ensuring that experts are fully engaged with the process and resolving any misunderstandings could be more difficult.

Adaptive methods of borrowing provide an alternative approach, in which the extent that borrowed data are down-weighted is influenced by disparity between the borrowed data and the observed data in the target subgroup. These methods were proposed for adaptive borrowing of external historical control data [21], and have also been discussed for borrowing information across strata or subgroups within trials [22, 23]. For example, a mixture prior with two components could be declared for the interaction term $\delta$ in the ODYSSEY trial, comprising one informative component based on expert opinion and one vague component. Weights chosen for the two components would control how quickly the data from older children were down-weighted in response to conflict between the borrowed and observed data. In the ODYSSEY trial, we assumed no conflict between borrowed and observed data, on the basis that the borrowed data were obtained within the same trial rather than from an external source. In addition, the interaction parameter is imprecisely estimated from the data so we would be very unlikely to observe conflict here. In a setting where the interaction parameter is better estimated from the data and conflict is observed between borrowed and observed information, it would be preferable to report results from the target subgroup data alone.

Feedback from the experts who provided opinions showed that they found stage 2 of the elicitation more difficult than the other two stages, because they found it hard to imagine failure rates not differing at all between DTG and SOC. A few experts commented that they would have liked additional information to inform their opinions, such as pharmacokinetic (PK) evidence or information about co-morbidities in the older and younger children. Preliminary PK data in ODYSSEY children weighing 6 to < 14 kg (*n* = 16) were presented to the 8 ODYSSEY investigators prior to the PENTA-ID meeting and ODYSSEY investigators were familiar with PK data in children weighing ≥ 14 kg. This evidence was not published at the time our elicitations were conducted, but in retrospect it would have been preferable to provide the preliminary results to all experts. We used a simple model and assumed normality for risk differences, in order to facilitate communication with clinicians. If normality were not considered appropriate, a more complicated model could be assumed; however, the correspondence between uncertainty about the interaction and the relative weights allocated to subgroups in the Bayesian analysis would then be lost. We considered it reasonable to assume normality for each expert’s probability distribution. Alternative nonparametric approaches to elicitation are available and would allow flexibility for the shape of the expert’s distribution [24]; these require additional quantities to be elicited and there is no particular rationale for their use in this setting.

## Conclusions

Borrowing information from a larger subgroup or subgroups can facilitate estimation of treatment effects in small patient groups within a clinical trial and lead to improved power and precision, as shown for the ODYSSEY trial. This approach can be beneficial in subgroups for which sufficient recruitment is difficult, and could potentially reduce the cost and duration of a trial and the risks and inconvenience to vulnerable participants. Informative prior distributions for interaction parameters are required to inform the degree of borrowing and can be informed by expert opinion, and we have demonstrated accessible methods for obtaining opinions.

## Supplementary Information

**Additional file 1: Figure S1. **Excel spreadsheet illustrating the correspondence between weights allocated to the data from older children and beliefs about the uncertainty range for the treatment difference in younger children. **Figure S2. **Drawing materials and counters provided to help experts visualise their probability beliefs.

## Data Availability

The example data sets generated and analysed during this research are available from the corresponding author on reasonable request.

## Abbreviations

AIDS: Acquired immune deficiency syndrome
ART: Antiretroviral therapy
CI: Confidence interval
CrI: Credible interval
DTG: Dolutegravir
HIV: Human immunodeficiency virus
PK: Pharmacokinetics
SOC: Standard of care
WHO: World Health Organization
