# Supplementary material for: Borrowing information across patient subgroups in clinical trials, with application to a paediatric trial
Source: BMC Med Res Methodol. 2022 Feb 20;22:49. doi: 10.1186/s12874-022-01539-3 (PMC8858505; doi:10.1186/s12874-022-01539-3)
Supplement: Supplementary file 1 — Additional file 1: Figure S1. Excel spreadsheet illustrating the correspondence between weights allocated to the data from older children and beliefs about the uncertainty range for the treatment difference in younger children. Figure S2. Drawing materials and counters provided to help experts visualise their probability beliefs. [file 12874_2022_1539_MOESM1_ESM.pdf]

Borrowing information across patient subgroups in clinical trials, with application to a paediatric trial: Additional material

Derivations for formulae (4) and (5)

Using only the data from the target subgroup, we would estimate the treatment effect θ_1 in the target subgroup as follows:

$$\theta_1 | y_1 \sim N(y_1, \sigma_1^2)$$

Using only the data from the larger subgroup, we would estimate θ_1 as follows:

$$\theta_1 | y_0, \delta \sim N\left(y_0, \sigma_0^2 + \sigma_\delta^2\right)$$

The joint likelihood function is then:

$$L(\theta_1; y_1, y_0, \delta) = \frac{1}{\sigma_1^2} exp\left\{\frac{(y_1 - \theta_1)^2}{2\sigma_1^2}\right\} \frac{1}{\sigma_0^2 + \sigma_\delta^2} exp\left\{\frac{(y_0 - \theta_1)^2}{2\left(\sigma_0^2 + \sigma_\delta^2\right)}\right\}$$

By differentiating the log likelihood and setting to 0, it can be shown that the maximum likelihood estimator of θ_1 is:

$$\widehat{\theta_1} = \frac{y_1/\sigma_1^2 + y_0/\left(\sigma_0^2 + \sigma_\delta^2\right)}{1/\sigma_1^2 + 1/\left(\sigma_0^2 + \sigma_\delta^2\right)}$$

with the following variance:

$$Var\left(\widehat{\theta_1}\right) = \frac{1}{1/\sigma_1^2 + 1/\left(\sigma_0^2 + \sigma_\delta^2\right)}$$

We have assumed a vague normal prior for θ_0: $\theta_0 \sim N(0, 10^6)$, and have assumed $\delta \sim N\left(0, \sigma_\delta^2\right)$ where σ_δ^2 is assumed fixed and known. The implied prior for $\theta_1 = \theta_0 + \delta$ is normal with zero mean and very large variance and therefore the posterior mean and variance approximate the likelihood-based estimate and variance. The posterior distribution for θ_1 is therefore (equation (4) in main text):

$$\theta_1 | y_1, y_0, \delta \sim N\left(\frac{y_1/\sigma_1^2 + y_0/\left(\sigma_0^2 + \sigma_\delta^2\right)}{1/\sigma_1^2 + 1/\left(\sigma_0^2 + \sigma_\delta^2\right)}, \frac{1}{1/\sigma_1^2 + 1/\left(\sigma_0^2 + \sigma_\delta^2\right)}\right)$$

The posterior mean is a weighted average of the estimated treatment effects y_1 and y_0 obtained for the target subgroup and larger subgroup respectively. We observe that the relative weight given to the larger subgroup is (equation (5) in main text):

$$\frac{1}{\sigma_0^2 + \sigma_\delta^2} \Big/ \left(\frac{1}{\sigma_1^2} + \frac{1}{\sigma_0^2 + \sigma_\delta^2}\right)$$

Full mathematical model (as presented descriptively in the main text)

$y_1 \sim N(\theta_1, \sigma_1^2)$, $y_0 \sim N(\theta_0, \sigma_0^2)$ where σ_1^2 and σ_0^2 are assumed fixed and known (estimated from data)

$\theta_1 = \theta_0 + \delta$, $\theta_0 \sim N(0, 10^6)$, $\delta \sim N\left(0, \sigma_\delta^2\right)$ where σ_δ^2 is assumed fixed and known (informed by elicited opinion)

$$\theta_1 | y_1, y_0, \delta \sim N\left(\frac{y_1/\sigma_1^2 + y_0/\left(\sigma_0^2 + \sigma_\delta^2\right)}{1/\sigma_1^2 + 1/\left(\sigma_0^2 + \sigma_\delta^2\right)}, \frac{1}{1/\sigma_1^2 + 1/\left(\sigma_0^2 + \sigma_\delta^2\right)}\right)$$

Materials used to support elicitations

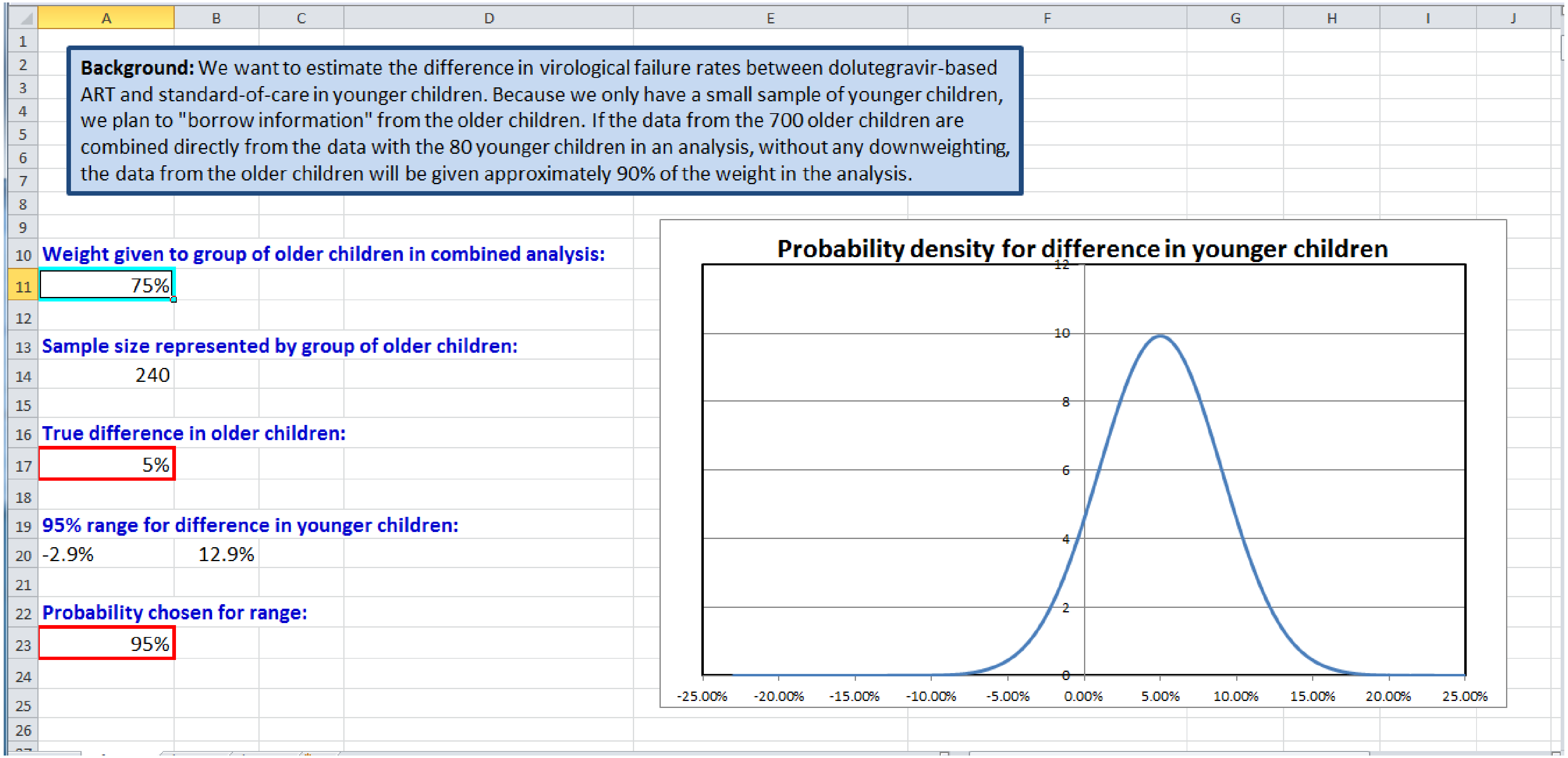

Figure S1 Excel spreadsheet illustrating the correspondence between weights allocated to the data from older children and beliefs about the uncertainty range for the treatment difference in younger children. Choices for the weight (blue cell), assumed treatment difference in older children, and the level of uncertainty assigned to the range (red cells) could be altered within the spreadsheet. In the spreadsheet, treatment differences were expressed as % benefit in favour of DTG to facilitate communication during elicitations, because it's easier to discuss changes to positive rather than negative values. (In the remainder of the paper, however, the sign is reversed and treatment differences are expressed as DTG – SOC.)

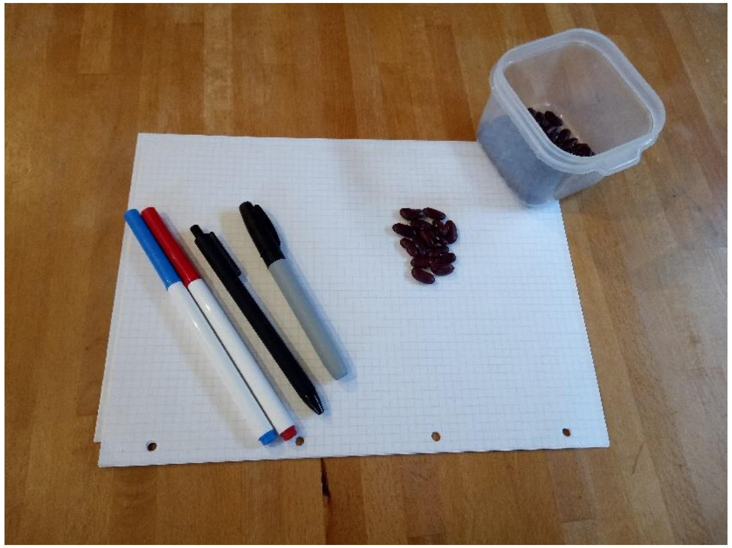

Figure S2 Drawing materials and counters provided to help experts visualise their probability beliefs.

ODYSSEY TRIAL TEAM

The ODYSSEY Trial Team consists of: (MRC CTU) Shabinah Ali, Abdel Babiker, Chiara Borg, Anne-Marie Borges Da Silva, Joanna Calvert, Deborah Ford, Joshua Gasa, Diana M. Gibb, Nasir Jamil, Sarah Lensen, Emma Little, Fatima Mohamed, Samuel Montero, Cecilia L. Moore, Rachel Oguntimehin, Anna Parker, Reena Patel, Tasmin Phillips, Tatiana Sarfati, Karen Scott, Clare Shakeshaft, Moira Spyer, Margaret Thomason, Anna Turkova, Rebecca Turner, Nadine Van Looy, Ellen White, Kaya Widuch, Helen Wilkes, Ben Wynne. **(PENTA-ID)** Carlo Giaquinto, Tiziana Grossele, Daniel Gomez-Pena, Davide Bilardi, Giulio Vecchia. **(INSERM-ANRS)** Alexandra Compagnucci, Yacine Saidi, Yoann Riault, Alexandra Coelho, Laura Picault, Christelle Kouakam. **(PHPT)** Tim R. Cressey, Suwalai Chalermpantmetagul, Dujrudee Chinwong, Gonzague Jourdain, Rukchanok Peongjakta, Pra-ornsuda Sukrakanchana, Wasna Sirirungsi. **(Sub-study Partners)** Janet Seeley, Sarah Bernays, Magda Conway, Nigel Klein, Eleni Nastouli, Anita De Rossi, Maria Angeles Munoz Fernandez, David Burger, Pauline Bollen, Angela Colbers, Hylke Waalewijn. **(Joint Clinical Research Centre, Uganda)** Cissy M. Kityo, Victor Musiime, Elizabeth Kaudha, Annet Nanduudu, Emmanuel Mujyambere, Paul Ocitti Labeja, Charity Nankunda, Juliet Ategeka, Peter Erim, Collin Makanga, Esther Nambi, Abbas Lugemwa, Lorna Atwine, Edridah Keminyeto, Deogratiuos Tukwasibwe, Shafic Makumbi, Emily Ninsiima, Mercy Tukamushaba, Rogers Ankunda, Ian Natuhurira, Miriam Kasozi, Baker Rubinga. **(Baylor College of Medicine Children's Foundation, Uganda)** Adeodata R. Kekitiinwa, Pauline Amuge, Dickson Bbuye, Justine Nalubwama, Winnie Akobye, Muzamil Nsibuka Kisekka, Anthony Kirabira, Gloria Ninsiima, Sylvia Namanda, Gerald Agaba, Immaculate Nagawa, Annet Nalugo, Florence Namuli, Rose Kadhuba, Rachael Namuddu, Lameck Kiyimba, Angella Baita, Eunice Atim, Olivia Kobusingye, Clementine Namajja, Africanus Byaruhanga, Rogers Besigye, Herbert Murungi, Geoffrey Onen. **(MUJHU Research Collaboration, Uganda)** Philippa Musoke, Linda Barlow-Mosha, Grace Ahimbisibwe, Rose Namwanje, Monica Etima, Mark Ssenyonga, Robert Serunjogi, Hajira Kataike, Richard Isabirye, Phionah Kibalama, Sarah Nakabuye, Barbara Musoke, David Balamusani, Monica Nolan. **(FAM-CRU, South Africa)** Mark F. Cotton, Anita Janse van Rensburg, Marlize Smuts, Catherine Andrea, Sumaya Dadan Sonja Pieterse, Vinesh Jaeven, Candice Makola, George Fourie, Kurt Smith, Els Dobbels, Peter Zuidewind, Hesti Van Huyssteen, Mornay Isaacs, Georgina Nentsa, Thabis Ncgaba, Candice MacDonald, Mandisa Mtshagi, Maria Bester, Wilma Orange, Ronelle Arendze, Mark Mulder, George Fourie. **(PHRU, South Africa)** Avy Violari, Nastassja Ramsagar, Afaaf Liberty, Ruth Mathiba, Lindiwe Maseko, Nakata Kekane, Busi Khumlo, Mirriam Khunene, Noshalaza Sbisi, Jackie Brown, Ryphina Madonsela, Nokuthula Mbadaliga, Zaakirah Essack, Reshma Lakha, Aasia Vadee, Derusha Frank, Nazim Akoojee, Maletsatsi Monametsi, Gladness Machache, Yolandie Fourie, Anusha Nanan-kanjee, Juan Erasmus, Angelous Mamiane, Tseleng Daniel, Fatima Mayat, Nomfundo Maduna, Patsy Baliram. **(Prapokklao Hospital, Thailand)** Chaiwat Ngampiyasakul, Pisut Greetanukroh, Wanna Chamjamrat, Praechadaporn Khannak. **(Phayao Hospital, Thailand)** Pornchai Techakunakorn, Thitiwat Thapwai, Patcharee Puangmalai, Ampai Maneekaew. **(Chiangrai Prachanukroh Hospital, Thailand)** Pradthana Ounchanum, Yupawan Thaweesombat, Areerat Kongponoi, Jutarat Thewsoongnoen. **(Nakornping Hospital, Thailand)** Suparat Kanjanavanit, Pacharaporn Yingyong, Thida Namwong, Rangwit Junkaew. **(Khon Kaen Hospital, Thailand)** Ussanee Srirompotong, Patamawadee Sudsaard, Siripun Nuanbuddee, Sookpanee Wimonklang. **(Mahasarakam Hospital, Thailand)** Sathaporn Na-Rajsima, Suchart_Thongpaen, Pattira Runarassamee, Watchara Meethaisong, Arttasid Udomvised. **(Klerksdorp Tshepong Hospital Complex, South Africa)** Ebrahim Variava, Modiehi Rakgokong, Dihedile Scheppers, Tumelo Moloantoa, Abdul Hamid Kaka, Tshepiso Masienyane, Akshmi Ori, Kgosimang Mmolawa, Pattamukkil Abraham. **(Durban International Clinical Research Site, South Africa)** Moherndran Archary, Rejoice Mosia, Sajeeda Mawlana, Rosie Mngqibisa, Rashina Nundlal, Elishka Singh, Penelope Madlala, Allemah Naidoo, Sphiwee Cebekhulu, Petronelle Casey, Collin

Pillay, Subashinie Sidhoo, Minenhle Chikowore, Lungile Nyantsa, Melisha Nunkoo, Terence Nair, Enbavani Pillay, Sheleika Singh, Sheroma Rajkumar. **(AHRI, South Africa)** Osee Behuhuma, Olivier Koole, Kristien Bird, Nomzamo Buthelezi, Mumsy Mthethwa. **(UZCRC, Zimbabwe)** James Hakim, Hilda Mujuru, Kusum Nathoo, Mutsa Bwakura-Dangarembizi, Ennie Chidziva, Shepherd Mudzingwa, Secrecy Gondo, Godfrey Musoro, Vivian Mumbiro, Gloria Tinago, Shirley Mutsai, Joy Chimanzi, Columbus Moyo, Ruth Nhema, Misheck Nkalo Phiri, Stuart Chitongo, Joshua Choga, Joyline Bhiri, Wilber Ishemunyoro, Makhosonke Ndlovu. **(HIVNAT, Thailand)** Thanyawee Puthanakit, Naruporn Kasipong, Sararut Chanthaburanun, Kesdao Nanthapisal, Thidarat Jupimai, Thornthun Noppakaorattanamanee, Torsak Bunupuradah, Wipaporn Natalie Songtaweesin, Chutima Saisaengjan. **(European Site Investigators)** Stephan Schultze-Straber, Christoph Konigs, Robin Kobbe, Felicia Mantkowski, Steve Welch, Jacqui Daglish, Laura Thrasyvoulou, Delane Singadia, Sophie Foxall, Judith Acero, Gosia Pasko-Szcech, Jacquie Flynn, Gareth Tudor-Williams, Farhana Abdulla, Srini Bandi, Jin Li, Sean O'Riordan, Dominique Barker, Richard Vowden, Colin Ball Eniola Nsirim, Kathleen McClughlin, India Garcia, Pablo Rojo Conejo, Cristina Epalza, Luis Prieto Tato, Maite Fernandez, Luis Escosa Garcia, Maria José Mellado Peña, Talia Sainz Costa, Claudia Fortuny Guasch, Antoni Noguera Julian, Carolina Estepa, Elena Bruno, Alba Murciano Cabeza, Maria Angeles Muñoz Fernandez, Paula Palau, Laura Marques, Carla Teixeira, Alexandre Fernandes, Rosita Nunes, Helena Nascimento, Andreia Padrao, Joana Tuna, Helena Ramos, Ana Constança Mendes, Helena Pinheiro, Ana Cristina Matos. **(Local Site Monitors)** Flavia Kyomuhendo, Sarah Nakalanzi, Cynthia Mukisa Williams, Ntombenhle Ngcobo, Deborah Pako, Jacky Crisp, Benedictor Dube, Precious Chandiwana, Winnie Gozhora. **(Independent Trial Steering Committee Members)** Ian Weller, Elaine Abrams, Tsitsi Apollo, Polly Clayden, Valériane Leroy. **(Independent Data Monitoring Committee Members)** Anton Pozniak, Jane Crawley, Rodolphe Thiébaut, Helen McIlleron. **(Endpoint Review Committee Members)** Alasdair Bamford, Hermione Lyall, Andrew Prendergast, Felicity Fitzgerald, Anna Goodman.
